# UHRF1 is a genome caretaker that facilitates the DNA damage response to γ-irradiation

**DOI:** 10.1186/2041-9414-1-7

**Published:** 2010-06-08

**Authors:** Helena Mistry, Laura Tamblyn, Hussein Butt, Daniel Sisgoreo, Aileen Gracias, Meghan Larin, Kalpana Gopalakrishnan, Manoor Prakash Hande, John Peter McPherson

**Affiliations:** 1 Department of Pharmacology and Toxicology, University of Toronto, Toronto, Ontario, M5 S 1A8, Canada; 2 Department of Physiology, Yong Loo Lin School of Medicine, National University of Singapore, 117597, Singapore

## Abstract

**Background:**

DNA double-strand breaks (DSBs) caused by ionizing radiation or by the stalling of DNA replication forks are among the most deleterious forms of DNA damage. The ability of cells to recognize and repair DSBs requires post-translational modifications to histones and other proteins that facilitate access to lesions in compacted chromatin, however our understanding of these processes remains incomplete. UHRF1 is an E3 ubiquitin ligase that has previously been linked to events that regulate chromatin remodeling and epigenetic maintenance. Previous studies have demonstrated that loss of UHRF1 increases the sensitivity of cells to DNA damage however the role of UHRF1 in this response is unclear.

**Results:**

We demonstrate that UHRF1 plays a critical role for facilitating the response to DSB damage caused by γ-irradiation. UHRF1-depleted cells exhibit increased sensitivity to γ-irradiation, suggesting a compromised cellular response to DSBs. UHRF1-depleted cells show impaired cell cycle arrest and an impaired accumulation of histone H2AX phosphorylation (γH2AX) in response to γ-irradiation compared to control cells. We also demonstrate that UHRF1 is required for genome integrity, in that UHRF1-depleted cells displayed an increased frequency of chromosomal aberrations compared to control cells.

**Conclusions:**

Our findings indicate a critical role for UHRF1 in maintenance of chromosome integrity and an optimal response to DSB damage.

## Background

UHRF1 (also known as Np95 and ICBP90) was originally identified as a protein whose subcellular expression pattern coincided with sites of DNA replication [1-3]. Further studies supported a role for this protein in S phase progression, particularly in replication of heterochromatin regions surrounding centromeres known as pericentric heterochromatin [4-6]. This role in heterochromatin replication and maintenance is linked to the ability of UHRF1 to facilitate several epigenetic modifications of histones and DNA [5-7]. UHRF1 binds to and ubiquitinates histone H3 [7,8] and facilitates deacetylation of lysine 8, 12, and 16 of heterochromatin histone H4 [6,9]. The SET and RING associated (SRA) domain of UHRF1 binds to hemi-methylated DNA and plays a crucial role in copying pre-existing methylation patterns onto newly replicated DNA by recruiting the DNA methyltransferase Dnmt1 to replication sites [10-14]. In addition to its function in duplicating DNA methylation patterns, UHRF1 binds to histone H3 tri-methylated at lysine 9 (H3K9me3) and plays a role in maintaining this histone modification in heterochromatin [7]. A recent study has pointed to the importance of a tandem tudor domain for UHRF1 binding to H3K9me3 [15].

Several studies have now identified a role for UHRF1 in the maintenance of heterochromatin modifications independent of its role in DNA replication. UHRF1 acts to facilitate promoter silencing [9,16-19]. This ability to repress transcription has been linked to UHRF1's recruitment of G9a histone methyltransferase and DNA methyltransferases Dnmt3a/b that repress transcription in euchromatin through the dimethylation of H3K9 and DNA methylation respectively [17,18].

Previous studies have demonstrated a critical role for UHRF1 in the cellular response to a wide range of stimuli that result in DNA damage. Murine embryonic stem cells with a targeted disruption in *Uhrf1 *are more sensitive to x-rays, UV light, base damaging agents and hydroxyurea than wild-type cells [20]. Ablation of human UHRF1 results in hypersensitivity to X-rays, UV light and hydroxyurea [21]. Despite these observations, the mechanism whereby UHRF1 confers a protective role to various genotoxic stresses remains unclear. In this study, we identify a novel role for this protein as a genome caretaker and demonstrate that the hypersensitivity of UHRF1-depleted cells to irradiation can be attributed to an impaired ability to mount an optimal DNA damage response.

## Materials and methods

### Cell lines

Human HeLa cells (ATCC) with diminished expression of UHRF1 were selected for resistance to puromycin following stable transfection with either HuSH 29mer short hairpin RNA (shRNA) constructs against UHRF1 (5'-AGG AGA CGT TCC AGT GTA TCT GCT GTC AG-3' and '5-TTC GTG GAC GAA GTC TTC AAG ATT GAG CG-3') or with a control shRNA plasmid (pRS-shGFP, non-effective, Origene), together with a vector conferring puromycin resistance. Three cloned cell lines expressing *UHRF1 *shRNA that demonstrated decreased levels of UHRF1, together with three cell lines transfected with control vector were selected for further analysis. Growth of individual cell lines was monitored by seeding cells at 3.5 × 10^5^/dish in triplicate and counting cell numbers using a hemacytometer at three day intervals. Growth curves show the cumulative mean cell number ± standard deviation of five counts.

### Western blotting

For Western analysis, cells were lysed in RIPA buffer (50 mM Tris-HCl pH 8.0, 150 mM NaCl, 1 mM PMSF, 1 mM EDTA, 1% Triton X-100, 1% sodium deoxycholate, 0.1% SDS, 5 μg/ml aprotinin, 5 μg/ml leupeptin, and 5 μg/ml pepstatin). Protein quantitation was determined by Bradford assay. Protein samples were resolved using 10% SDS-PAGE gels and transferred onto PVDF membrane overnight at 4°C. Membranes were blocked in Tris Buffered Saline with 5% milk and 0.1% Tween for 1 h at room temperature. Membranes were immunoblotted with the following primary antibodies diluted in Tris Buffered Saline with 5% milk and 0.1% Tween overnight at 4°C: polyclonal anti-tubulin (Sigma), monoclonal anti-UHRF1 (BD Bioscience) followed by 1 h incubation at room temperature with the appropriate secondary antibody: anti-rabbit HRP-linked IgG or anti-mouse HRP-linked IgG (GE Healthcare). Protein detection was performed using the ECL Western blotting detection system (GE Healthcare) and exposed to scientific imaging film (Bioflex).

### Indirect Immunofluorescence and micronuclei analysis

HeLa cells were seeded onto coverslips (5 × 10^4^/coverslip) pre-coated with 1% gelatin and 1% BSA. Following fixation in methanol/acetone and permeabilization with 0.4% Triton-X in PBS for 20 min, cells were blocked (1% donkey serum/0.2% Triton-X) for 20 min and then incubated overnight with either anti-histone H3 trimethylated on lysine 9 (anti-H3K9me3) or anti-histone H4 trimethylated on lysine 20 (anti-H4K20me3) overnight at 4°C (Millipore). Cells were then incubated with TRITC-conjugated secondary antibodies, counterstained with 4,6-diamidino-2-phenylindole (DAPI) and mounted with Vectashield (Vector laboratories). Images were acquired using an Imager.Z1 epifluorescence microscope and Axiovision software (Zeiss) following deconvolution. For micronuclei analysis, three UHRF1-depleted cell lines and three control cell lines were scored for the presence of micronuclei in triplicate, with each determination scoring > 200 cells.

### Clonogenic assay

Fixed amounts of cells exposed to varying doses of γ-irradiation (Nordion Gamma-cell, Ontario Cancer Institute) were seeded in 60 mm dishes in Dulbecco's Minimum Essential Media supplemented with 10% fetal bovine serum (Invitrogen). Cells were left to form colonies for seven days. Colonies were fixed and stained with methylene blue in methanol. All survival curves were produced from an average ± standard deviation of three to six determinations and are presented as a percent of control (non-irradiated) cells.

### Flow cytometry

Cell-cycle analysis by bromodeoxyuridine (BrdU) and propidium iodide (PI) double staining was conducted essentially as described previously [22]. Cells (5 × 10^5^/100 mm dish) exposed to 0, 1 or 5 Gy were collected and fixed in 70% ethanol at 0, 1, 3, 6, 12 or 24 h after exposure and stored at -20°C prior to analysis. Measurement of γH2AX and PI double staining by flow cytometry was performed as described previously, but with the following modifications [22]. Cells were fixed in ice-cold 70% ethanol for 20 min, rinsed in PBS and gently vortexed in PBS + 0.4% TritonX for 15 min, then rinsed in PBS. Cells were then incubated with anti-γH2AX (1:200 of 05-636, Millipore) in the dark for 3 h at room temperature in 0.2% TritonX/1% donkey serum. After 1 rinse in PBS, cells were then incubated in anti-mouse FITC (1:200, Jackson ImmunoResearch Labs) for 30 min at room temperature. After 1 rinse in PBS, cells were stained in PI (50 μg/ml in PBS) for 30 min at room temperature, then analyzed by flow cytometry.

### Karyotype analysis

FISH and its subsequent analysis were performed as described before [23]. Slides hydrated in PBS were fixed in 4% v/v formaldehyde: PBS, washed 3 × 5 min in PBS, treated with 0.1 mg/mL pepsin (P-70000, *Sigma*) at pH 2 (3 min for fibroblasts, 1 1/2 min for lymphoblastoids), followed by repeated formaldehyde fixation and PBS washes before dehydration in an ethanol series (70%, 90% and 100%). Air-dried slides were first denatured at 80°C for 3 min with hybridization mixture containing deionized formamide (F9037; *Sigma*), 0.5 μg/mL Cy-3-conjugated-(CCCTAA)_3 _PNA probe complementary to telomeric sequence and 3 μg/mL Fluorescein isothiocyanate (FITC)-conjugated-centromic PNA probe (Applied Biosystems) in 10 mM Tris (pH 7), then hybridized in the dark for 2 h at room temperature. Slides were then washed 2 × 15 min in 70% v/v formamide (Merck)/1% w/v BSA/10 mM Tris (pH 7.4) and 3 × 5 minute in 0.1 M Tris (1^st ^Base, Singapore)/0.15 M NaCl (pH 7.2)/0.08% v/v Tween 20 (Sigma). Slides were dehydrated in an ethanol series, air-dried in the dark and counterstained with 0.0375 μg/mL DAPI in mounting media (Vectashield; Vector Laboratories). Images from approximately 50 metaphases were captured using an Axioplan 2 imaging fluorescent microscope (Zeiss) and analyzed for chromosomal aberrations such as breaks or fusions, which are indicative of genomic instability, with Isis Imaging Software (Metasystems, Germany).

## Results

### Establishment of UHRF1-depleted cell lines

**Figure 1:**
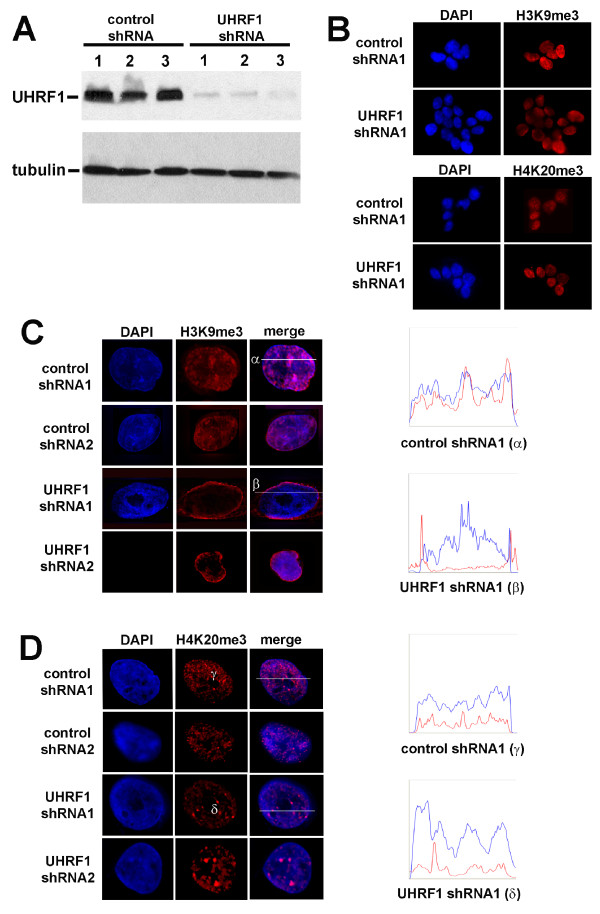
**Characterization of UHRF1-depleted HeLa cell lines**. (**A**) Stable depletion of UHRF1 by shRNA in HeLa cells as shown by Western analysis (UHRF1 shRNA clones 1-3) compared to clones expressing a nonspecific shRNA (control shRNA clones 1-3). Tubulin levels are shown as loading controls. (B) Representative indirect immunofluorescence of H3K9me3 and H4K20me3 in control and UHRF1 shRNA cells at 40 × magnification. (**C-D**) Representative indirect immunofluorescence deconvolution of H3K9me3 and H4K20me3 in two UHRF1 shRNA and two control shRNA cell lines. Line traces are shown on the right (blue = DAPI, red = H3K9me3 or H3K20me3) for representative photomicrographs labeled with Greek characters.

To investigate the requirement of UHRF1 in genome integrity, we derived stable clones of HeLa cells expressing shRNA targeted to UHRF1. Three clones were selected that exhibited dramatically reduced UHRF1 expression, as demonstrated by Western analysis with anti-UHRF1 (Figure 1A). Cells expressing the first UHRF1 shRNA (UHRF1 shRNA cell line 1 and 2) or the second UHRF1 shRNA (cell line 3) showed a drastic depletion of UHRF1 protein levels compared to the three cell lines expressing control shRNA. Previous studies have demonstrated a role for UHRF1 in maintenance of heterochromatin organization and replication [6,7,24]. To examine the heterochromatin status in UHRF1-depleted cells, we immunostained UHRF1-depleted and control cells with antibodies against heterochromatin markers H3K9me3 and H4K20me3. Control cell lines exhibited focal nuclear staining of H3K9me3 and H4K20me3 that superimposed DAPI-dense regions of nuclear chromatin that represent heterochromatin (Figure 1B-D). In marked contrast, UHRF1-depleted cells showed loss of focal H3K9me3 accumulation, with H3K9me3 staining redistributed at the nuclear periphery (Figure 1C). The impact on H3K9me3 did not appear to reflect a generalized disruption of heterochromatin structure, as UHRF1-depleted cells displayed focal staining of H4K20me3 that was equivalent to that observed in control shRNA cell lines, although H4K20me3 appeared to be localized in larger focal regions than in control cells (Figure 1D). In both control and UHRF1-depleted lines, no fluctuation in either H3K9me3 or H4K20me3 staining was observed as a consequence of cell cycle position. Taken together, these findings demonstrate a significant reorganization of heterochromatin in cells depleted of UHRF1.

### Impact on proliferation and susceptibility to irradiation

**Figure 2:**
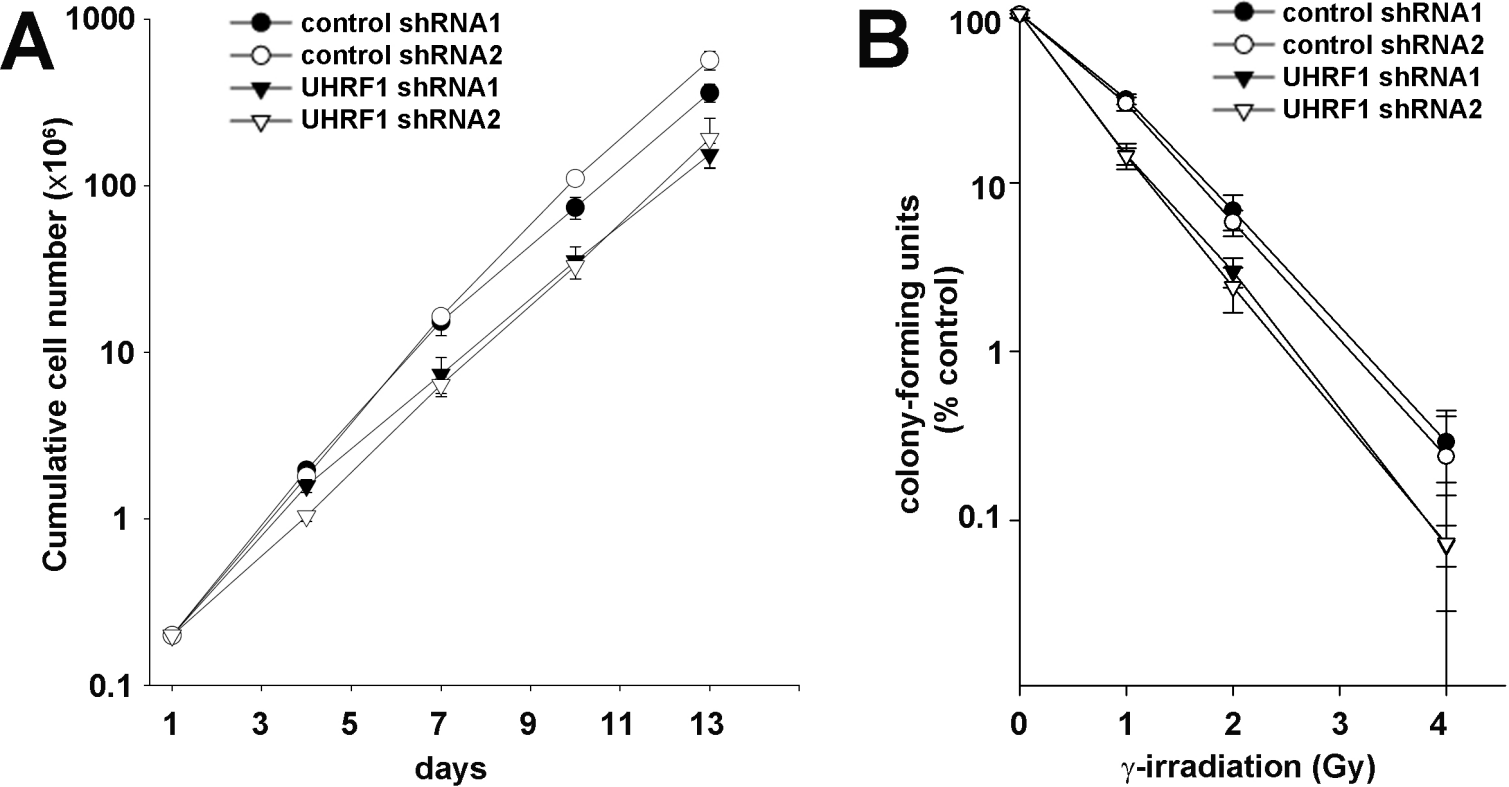
**Impact of UHRF1 loss on proliferation and sensitivity to γ-irradiation**. (**A**) Cell growth curves of UHRF1-depleted and control cell lines. (**B**) Susceptibility of UHRF1-depleted cell lines to γ-irradiation. Cell lines were exposed to ionizing radiation and sensitivity was measured by colony formation.

Previous studies have documented that depletion of UHRF1 results in proliferation arrest [5,25]. To assess whether reducing levels of UHRF1 would impact cell proliferation, we generated cell growth curves for UHRF1 depleted and control lines (Figure 2A). UHRF1-depleted cells proliferated at a slightly slower rate than control cell lines, an effect that was only discernible following an extended (> 1 week) of analysis. Previous studies using murine embryonic stem cells with a targeted disruption of *Uhrf1 *and human cells with reduced UHRF1 have shown that these cells are more sensitive to γ-irradiation as analyzed by clonogenic assay [20,21]. To confirm these findings, two cell lines expressing UHRF1 shRNAs and two cell lines expressing control shRNAs were analyzed for sensitivity to γ-irradiation by clonogenic assay. As expected, cell lines depleted for UHRF1 exhibited hypersensitivity to γ-irradiation compared to control cell lines (Figure 2B).

### Cell cycle analysis following irradiation

**Figure 3:**
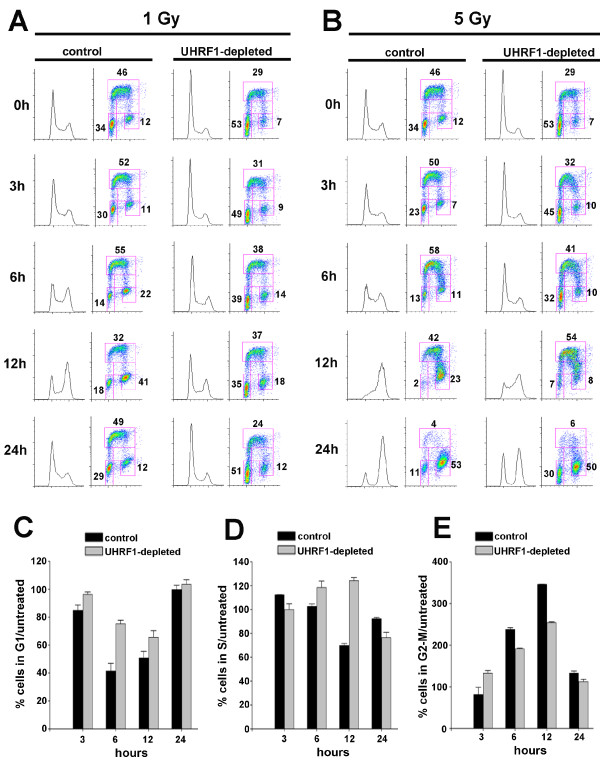
**Cell cycle analysis in UHRF1-depleted cells following γ-irradiation**. UHRF1 or control shRNA-expressing cells were pulsed with BrdU and then treated with 1 Gy (**A**) or 5 Gy (**B**) of γ-irradiation. Cells were harvested at the indicated times and stained with PI and for BrdU. For each time point, representative DNA content histograms (PI stain) are shown on the left with corresponding BrdU-PI bivariate plots on the right. Numerical values indicate percentage of G1, S and G2/M cells for each time point. The percentage change of cells in either G1 phase (**C**), S phase (**D**) or G2/M phases (**E**) compared to non-irradiated cells are plotted as mean ± S.D. of three independent experiments (shown for 1 Gy).

To monitor capacity for cell cycle arrest following DNA damage, we examined the percentage of cells at various stages of the cell cycle for one UHRF1 shRNA cell line and one control shRNA cell line following γ-irradiation. Cells were collected at various times after radiation (1 or 5 Gy) for cell cycle analysis following staining with anti-BrdU and PI. In the absence of irradiation, UHRF1-depleted cells showed a higher percentage of cells within the G1 phase fraction and a decrease in the S phase fraction compared to control-depleted cells (Figure 3A and 3B). Following 1 Gy of irradiation, controls exhibited a transient arrest in cell cycle progression with an accumulation of cells in the G2/M fraction, a response typical of HeLa cells. For control cells, this transient arrest was maximal 12 h post-irradiation, with a nearly four-fold increase in cell accumulation in the G2/M fraction compared to non-irradiated cells (Figure 3A, C-E). In contrast, UHRF1-depleted cells showed a decreased tendency to arrest following irradiation, with only a two-fold increase in the G2/M fraction and no noticeable decline in the S phase fraction 12 h after irradiation compared to non-irradiated cells. By 24 h, cell cycle progression resumed for both UHRF1-depleted cells and controls. In contrast, the majority of cells irradiated with 5 Gy exhibited a more drastic arrest in cell cycle progression leading to a predominant accumulation of cells in the G2/M fraction, (Figure 3B). At 12 h following 5 Gy, control cells accumulated in the G2/M fraction compared to earlier time points; howver, UHRF1-depleted cells showed a delayed accumulation in G2/M compared to controls (Figure 3B).

### γH2AX formation following γ-irradiation

**Figure 4:**
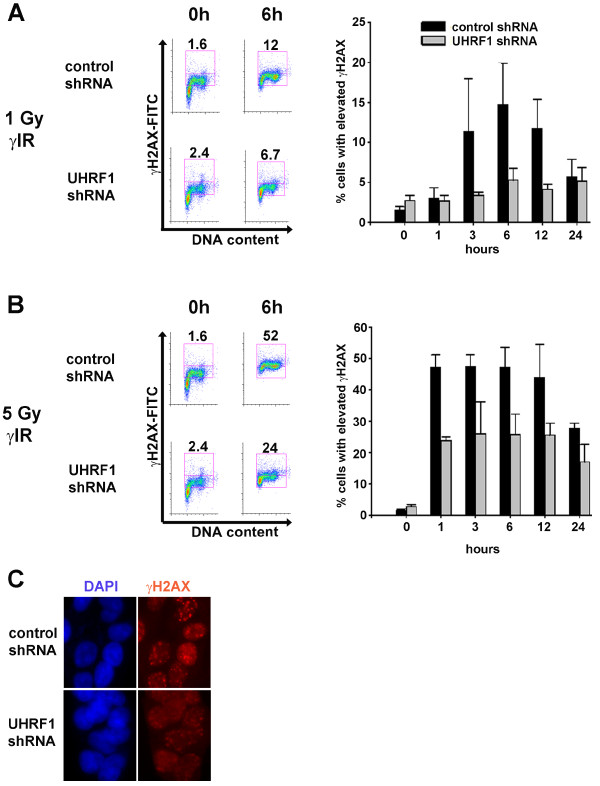
**Decreased irradiation-induced γH2AX in UHRF1-depleted cells**. UHRF1 and control shRNA-expressing cells were exposed to either 1 Gy (**A**) or 5 Gy (**B**) γ-irradiation and harvested following 0, 1, 3, 6, 12 or 24 h. Representative histograms plot γH2AX expression as measured by γH2AX-FITC intensity/cell (*y*-axis) vs. DNA content (*x*-axis) (**left hand side of panel**). Numbers indicate the percentage of cells showing elevated γH2AX levels. The percentage of cells with elevated γH2AX expression plotted is the mean ± S.D. of three independent experiments (**right hand side of panel**). (**C**) The decreased accumulation of γH2AX in UHRF1-depleted cells was confirmed by indirect immunofluorescence 12 h after 5 Gy γ-irradiation.

Phosphorylation of H2AX on serine 139 (γH2AX) is an early event after introduction of DSBs [26]. To monitor the kinetics and extent of γH2AX following irradiation, we measured γH2AX content 0, 1, 3, 6, 12 and 24 h in controls and UHRF1-depleted cells following 1 Gy or 5 Gy of γ-irradiation (Figure 4). Non-irradiated cells depleted of UHRF1 showed a nominally higher percentage of cells positive for γH2AX compared to control cells. Following 1 Gy of exposure, control cells showed a dramatic increase in the percentage of cells positive for γH2AX as expected, with maximal levels at 6 h that decreased to a level similar to non-irradiated cells by 24 h (Figure 4A). This time course of γH2AX induction corresponded to the degree of cell cycle arrest observed at 1 Gy (Figure 3A). Interestingly, the percentage of cells positive for γH2AX was markedly lower when UHRF1 was depleted compared to control cells. Following exposure to 5 Gy of irradiation, control cells showed a more drastic increase of γH2AX-positive cells with maximal levels 1-6 h following irradiation. Again, UHRF1-depleted cells showed an attenuated increase in the percentage of γH2AX-positive cells (Figure 4B). These findings were confirmed by detection of γH2AX by indirect immunofluorescence (Figure 4C). Taken together, these findings suggest that accumulation of γH2AX is defective in UHRF1-depleted cells following γ-irradiation.

### Micronuclei, centrosome copy number and karyotype

**Figure 5:**
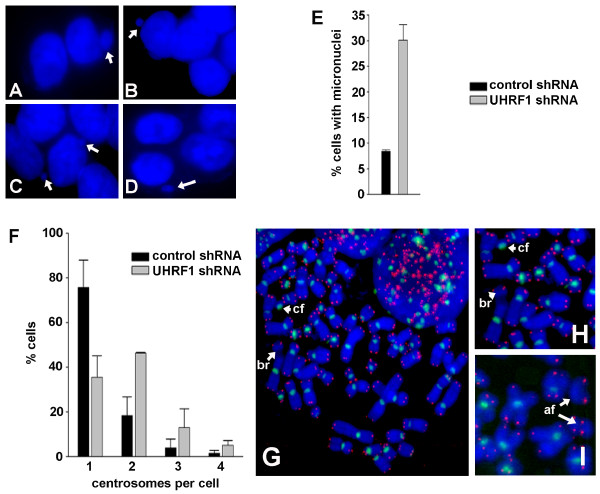
**Chromosomal instability in UHRF1-depleted cells**. (**A-D**) Representative photomicrographs showing presence of micronuclei in UHRF1-depleted cells. (**E**) Percentage of cells with micronuclei in control cells vs. UHRF1-depleted cells. Determinations from each cell line were performed in triplicate (n ≥ 200 cells/determination) and error bars represent standard deviations from the mean. (**F**) Percentage of control or UHRF1-depleted cells with 1, 2, 3, or 4 centrosomes per cell. (**G-I**) Representative images of DAPI-stained metaphases from UHRF1-depleted cells showing chromosomal aberrations (image shown in **H **is magnification of image in **G**). br = chromosome break, cf = centrosomal fragment, af = acentric fragments. Telomeres are visualized in red, centromeres in green.

Given the compromised DNA damage response in UHRF1-depleted cells, we sought to determine whether UHRF1 depletion conferred chromosomal instability. We stained three UHRF1-depleted and three control cell lines with DAPI and scored incidence of micronuclei (Figure 5A-D). Micronuclei are cytoplasmic chromosome fragments that fail to segregate properly during mitosis and are excluded from daughter nuclei at telophase [27,28]. Compared to the three control shRNA cell lines, UHRF1 shRNA cell lines exhibited a higher incidence of micronuclei (Figure 5E). We sought to determine whether UHRF1 impacts centrosome status in control and UHRF1-depleted cells. Examination of centrosome copy number following immunostaining with γ-tubulin revealed that the frequency of UHRF1-depleted cells with 1 centrosome per cell was lower compared to control cells and the frequency of UHRF1-depleted cells with 2 or more centrosomes per cells was higher compared to control cells (Figure 5F). To further assess alterations in chromosome integrity, we conducted karyotypic analyses on UHRF1-depleted and control cells. Visualization of telomeres and centromeres during karyotyping further clarified the scoring of aberrant metaphase chromosomes. Consistent with our observations of micronuclei and centrosomes, metaphase karyotypes of UHRF1-depleted cells showed a dramatic increase in the number of cells containing chromosome aberrations, particularly chromosome fragments (Figure 5G-I and Table 1).

**Table 1:** Increased chromosomal instability in UHRF1-depleted cells^a^.

**Cell lin**e	Chromosome Breaks	Chromatid Breaks	Fusions	Fragments	Total No. of aberrations
Control shRNA-1	0	0	1 (0.02/cell)	8 (0.16/cell)	9 (0.18/cell)
Control shRNA-2	0	0	2 (0.04/cell)	12 (0.24/cell)	14 (0.28/cell)
Control shRNA-3	2 (0.04/cell)	0	3 (0.06/cell)	10 (0.2/cell)	15 (0.3/cell)
UHRF1 shRNA-1	1 (0.02/cell)	0	5 (0.1/cell)	47 (0.94/cell)	53 (1.06/cell)
UHRF1 shRNA-2	1 (0.02/cell)	0	5 (0.1/cell)	51 (1.02/cell)	57 (1.14/cell)
UHRF1 shRNA-3	9 (0.18/cell)	1 (0.02/cell)	1 (0.02/cell)	54 (1.08/cell)	65 (1.3/cell)

^a^Results shown are number of chromosomal abnormalities detected in fifty metaphase spreads from three control shRNA and three UHRF1 shRNA (UHRF1-depleted) cell lines. The frequency of a given abnormality/metaphase is shown below in parentheses.

## Discussion

Using cell lines designed to permanently express reduced levels of UHRF1, we have demonstrated that UHRF1 is essential for maintaining the DNA damage response and genome integrity. Depletion of UHRF1 resulted in a greater propensity for cells to exhibit chromosomal instability, as shown by the increased frequency of micronuclei, supernumerary centrosomes and chromosome fragments in UHRF1-depleted cells. UHRF1 depleted cells exhibit increased susceptibility to DSB damage, compromised ability to undergo cell cycle arrest following DSB damage and decreased ability to accumulate γH2AX.

Pericentric heterochromatin is the highly compacted chromatin surrounding the centromere, a region that is easily detected as DAPI-dense chromocenters in certain cell lines. Recent studies have demonstrated several important mechanisms whereby UHRF1 contributes to the maintenance of heterochromatin. DNA replication of heterochromatin presents several challenges, as the cell replicates not only DNA but also the spatial organization of compacted chromatin regions. Heterochromatin is characterized by hypermethylation of DNA and an increased accumulation in certain post-translational modifications to histones such as tri-methylation of histones H3 and H4 at lysines 9 and 20 respectively. UHRF1 is now considered to be a crucial component of the protein machinery that ensures heterochromatin epigenetic markers are recreated on newly replicated strands of DNA. UHRF1 plays an integral role in regenerating methylation of newly-replicated DNA by recruiting the DNA methyltransferase DMNT1 [10,11]. In addition, UHRF1 also binds to H3K9me3 which are highly represented in heterochromatin. Recent studies have reported conflicting results on the requirements of UHRF1 for maintaining H3K9me3 in heterochromatin. One report states that the heterochromatic distribution of H3K9me3 in cells with stable depletion of UHRF1 by shRNA was disrupted, with depleted cells showing diffuse H3K9me3 staining compared to the focal heterochromatic enrichment in control cells [7]. Our observations confirm this finding, but contrast a subsequent study that does not report any significant changes in the redistribution of heterochromatin markers such as H3K9me3 in NIH 3T3 cells with UHRF1 transiently reduced by siRNA [24]. We suggest that the discrepancy may be due to experimental methodology employed, in that perhaps a transient reduction in UHRF1 levels will not impact heterochromatin histone marker modifications to the same extent as long term depletion of UHRF1.

We observed a slight impediment in the proliferation ability of UHRF1-depleted cells compared to control cell lines, in keeping with observations from previous studies [5,9,17,25]. The impact of loss of UHRF1 on proliferative capacity of cells appears to vary on the cell model used. UHRF1 depletion may impact S phase progression due to a compromised ability to replicate heterochromatin or through reversal of repressed expression of factors that impede S phase entry, such as p21 [17]. Accordingly, the overall effects of UHRF1 on proliferation could vary in a cell-type dependent manner on the degree of heterochromatin compaction and distribution.

Our analysis of non-irradiated UHRF1-depleted cells revealed a decreased fraction of cells in S phase with a corresponding increased fraction in G1 (not shown) compared to control cells. This decreased tendency for cells to be in S phase in the absence of UHRF1 has been observed in prior studies [5,9,25]. A previous report observed that loss of UHRF1 led a small fraction of cells to be accumulated in G1 when challenged with doxorubicin, leading to the conclusion that loss of UHRF1 unmasked a G1 checkpoint response to DNA damage [4]. Although we see a similar increase in the G1 peak of cells exposed to irradiation, we attribute this to the cell cycle profile that the UHRF1-depleted cells demonstrate prior to irradiation, and not as the result of a novel checkpoint response to DNA damage.

Given the role of UHRF1 in maintenance of heterochromatin structure and our findings that loss of UHRF1 compromises chromosomal integrity, we propose that the genomic instability observed in UHRF1-depleted cells is likely due to disruption of heterochromatin structure, particularly the heterochromatin surrounding centromeres known as pericentric heterochromatin. Previous studies have determined that maintenance of the stringent pericentric heterochromatin structure is required to protect genomic stability [29,30]. In particular, loss of H3K9me3 in this form of heterochromatin by the ablation of H3K9 methyltransferases Suv39h1 and Suv39h2 is sufficient to compromise chromosome segregation that could lead to the chromosomal aberrations seen in UHRF1-depleted cells [30].

A variety of studies have shown that loss of UHRF1 activity leads to the sensitization of cells to a variety of genotoxic agents including γ-irradiation, however the reason for this sensitivity has not been addressed [20,21,25]. Here we have demonstrated UHRF1-depleted cells exhibit an impaired ability to accumulate γH2AX following irradiation. Surprisingly, these cells show a reduced ability to generate γH2AX, a hallmark signal of DSB repair despite the observation that loss of UHRF1 increases chromosomal instability and DSBs. Given the role of UHRF1 in heterochromatin maintenance and replication, our findings suggest that the chromatin modification properties of UHRF1 in heterochromatin are required for an optimal response to DSB damage caused by γ-irradiation. Approximately 10% of all nucleosomes in mammalian chromatin contain the histone H2A variant H2AX. Following the initiation of DSB damage by irradiation, the Mre11-Rad50-NBS1 (MRN) complex binds to free DNA ends and recruits PIK-family protein kinases such as ATM, ATR or DNA protein kinase that cause the initial phosphorylation of H2AX on serine 139 (γH2AX). The recruitment of additional activated ATM to DSBs by the γH2AX-binding protein MDC1 serves to propagate the signal and amplify the focal accumulation of γH2AX, which then serves as a beacon for other DNA repair factors [reviewed in [31,32]].

Structural barriers of chromatin must be transiently removed during processing of DNA damage and restored following repair. Our findings of decreased γH2AX in UHRF1-depleted cells suggest that the disruption of heterochromatin associated with loss of UHRF1 compromises the accessibility of DNA damage response factors to process and repair DSBs. Reducing the degree of chromatin compaction through the reduction of histone H1 levels or inhibition of histone deacetylase activity has been demonstrated to increase the strength of the DNA damage response by increasing the extent of signaling generated per DNA lesion [33]. The focal phosphorylation of H2AX following γ-irradiation has been shown to occur less efficiently in heterochromatin compared to euchromatin, suggesting that changes in the packaging properties of chromatin have a marked impact on the strength of γH2AX signal generated in cells [34,35].

A recent study using an *in vitro *approach reported that UHRF1 can facilitate access of modifying enzymes to DNA encased in nucleosomal arrays [24]. We propose that UHRF1 may allow changes in heterochromatin structure that permit access of DNA surveillance and repair enzymes and that loss of this property may be responsible for the impaired DNA damage response observed in cells with reduced UHRF1. For example, our group and others show that stable reduction in UHRF1 does not change the total amount of H3K9me3 in cells but disrupts the focal heterochromatin enrichment of H3K9me3 normally observed in cells [7]. The methylation status of histone H3 on lysine 9 has not been reported to fluctuate in response to DNA damage, but serves as a binding scaffold for several heterochromatin proteins such as members of the HP1 family that have recently been implicated in the DNA damage response [36-38]. Release of HP-1β bound to chromatin by disruption of its H3K9me-associated binding domain facilitates formation of γH2AX foci and loss of HP1-β has been shown to result increased chromosomal instability [36,39]. In contrast, heterochromatin proteins HP1-α, HP1-β, and HP1-γ were shown to be recruited and accumulate at sites of DNA damage in a manner that is independent of H3K9me3 status [38]. Changes in heterochromatin modifications such as H3K9me3 and HP1 binding distribution could impact other factors that bind to HP1 proteins such as KAP-1, a protein recently shown to facilitate the ATM-mediated repair of DSBs in heterochromatin [37,40] or the histone acetyltransferase Tip60, which is activated by association with H3K9me3 [41]. Given that the extent and distribution of H3K9 methylation differs among cell lineages and is altered in human tumours, changes in methylation signatures of heterochromatin may contribute to the extent of genomic instability in tumour cells or to the therapeutic efficacy of antineoplastics by impacting the efficacy of DNA repair at specific chromatin regions in a cell-type specific manner [42,43].

## List of Abbreviations

**DAPI: **4,6-diamidino-2-phenylindole; **γH2AX: **histone H2AX phosphorylated on serine 139; **H3K9me3**: tri-methylated lysine 9 of histone H3; **H4K20me3**: tri-methylated lysine 20 on histone H4; **HP1**: heterochromatin protein 1; **UHRF1**: Ubiquitin-like, containing PHD and RING finger domains, 1.

## Competing interests

The authors declare that they have no competing interests.

## Authors' contributions

HM generated the cell lines, performed the clonogenic assay and indirect immunofluorescence with DS LT performed the FACS analysis. HB and AG performed the Western analysis and assisted with the indirect immunofluorescence. DS performed the micronuclei and centrosome analysis. ML generated growth curve and assisted with the clonogenic assay. KG and MPH performed the karyotyping analysis. JPM conceived the study, participated in its design and coordination and wrote the manuscript. All authors read and approved the final manuscript.

## References

[B1] Muto M Utsuyama M Horiguchi T Kubo E Sado T Hirokawa K The characterization of the monoclonal antibody Th-10a, specific for a nuclear protein appearing in the S phase of the cell cycle in normal thymocytes and its upregulated expression in lymphoma cell lines Cell Proliferation 1995 28 645 657 10.1111/j.1365-2184.1995.tb00051.x 8634372

[B2] Fujimori A Matsuda Y Takemoto Y Hashimoto Y Kubo E Araki R Fukumura R Mita K Tatsumi K Muto M Cloning and mapping of NP95 gene which encodes a novel nuclear protein associated with cellular proliferation Mamm Genome 1998 9 1032 1035 10.1007/s003359900920 9880673

[B3] Uemura T Kubo E Kanaari Y Ikemura T Tatsumi K Muto M Temporal and spatial localization of novel nuclear protein NP95 in mitotic and meiotic cells Cell Structure and Function 2000 25 149 159 10.1247/csf.25.149 10984098

[B4] Arima Y Hirota T Bronner C Mousli M Fujiwara T Niwa S-i Ishikawa H Saya H Down-regulation of nuclear protein ICBP90 by p53/p21-dependent DNA-damage checkpoint signals contributes to cell cycle arrest at G1/S transition Genes to Cells 2004 9 131 142 10.1111/j.1356-9597.2004.00710.x 15009091

[B5] Bonapace IM Latella L Papait R Nicassio F Sacco A Muto M Crescenzi M Di Fiore PP Np95 is regulated by E1A during mitotic reactivation of terminally differentiated cells and is essential for S phase entry J Cell Biol 2002 157 909 914 10.1083/jcb.200201025 12058012 PMC2174046

[B6] Papait R Pistore C Negri D Pecoraro D Cantarini L Bonapace IM Np95 is implicated in pericentromeric Heterochromatin replication and in major satellite silencing Mol Biol Cell 2007 18 1098 1106 10.1091/mbc.E06-09-0874 17182844 PMC1805105

[B7] Karagianni P Amazit L Qin J Wong J ICBP90, a novel methyl K9 H3 binding protein linking protein ubiquitination with heterochromatin formation Mol Cell Biol 2008 28 705 717 10.1128/MCB.01598-07 17967883 PMC2223417

[B8] Citterio E Papait R Nicassio F Vecchi M Gomiero P Mantovani R Di Fiore PP Bonapace IM Np95 is a histone-binding protein endowed with ubiquitin ligase activity Mol Cell Biol 2004 24 2526 2535 10.1128/MCB.24.6.2526-2535.2004 14993289 PMC355858

[B9] Unoki M Nishidate T Nakamura Y ICBP90, an E2F-1 target, recruits HDAC1 and binds to methyl-CpG through its SRA domain Oncogene 2004 23 7601 7610 10.1038/sj.onc.1208053 15361834

[B10] Bostick M Kim JK Esteve P-O Clark A Pradham S Jacobsen SE UHFR1 plays a role in maintaining DNA methylation in mammalian cells Science 2007 317 1760 1764 10.1126/science.1147939 17673620

[B11] Sharif J Muto M Takebayashi S-I Suetake I Iwamatsu A Endo TA Shinga J Mizutani-Koseki Y Toyoda T Okamura K Tajima S Mitsuya K Okano M Koseki H The SRA protein Np95 mediates epigenetic inheritance by recruiting Dnmt1 to methylated DNA Nature 2007 450 908 912 10.1038/nature06397 17994007

[B12] Arita K Ariyoshi M Tochio H Nakamura Y Shirakawa M Recognition of hemi-methylated DNA by the SRA protein UHRF1 by a base-flipping mechanism Nature 2008 455 818 821 10.1038/nature07249 18772891

[B13] Avvakumov GV Walker JR Xue S Li Y Duan S Bronner C Arrowsmith CH Dhe-Paganon S Structural basis for recognition of hemi-methylated DNA by the SRA domains of human UHRF1 Nature 2008 455 822 825 10.1038/nature07273 18772889

[B14] Qian C Li S Jakonic J Zeng L Walsh MJ Zhou MM Structure and hemi-methylated CpG binding of the SRA domain from human UHRF1 J Biol Chem 2008 283 34490 34494 10.1074/jbc.C800169200 18945682 PMC2596396

[B15] Rottach A Frauer C Pichler G Bonapace IM Spada F Leonhardt H The multi-domain protein Np95 connects DNA methylation and histone modification Nucl Acids Res 2010 38 6 1796 804 10.1093/nar/gkp1152 20026581 PMC2847221

[B16] Achour M Jacq X Ronde P Alhosin M Charlot C Jeanblanc M Macaluso M Giordano A Hughes AD Schini-Kerth VB Bronner C The interaction of the SRA domain of ICBP90 with a novel domain of DNMT1 is involved in the regulation of VEGF gene expression Oncogene 2008 27 2187 2197 10.1038/sj.onc.1210855 17934516

[B17] Kim JK Esteve PO Jacobsen SE Pradham S Uhrf1 binds G9a and participates in p21 transcriptional regulation in mammalian cells Nucl Acids Res 2008 37 493 505 10.1093/nar/gkn961 19056828 PMC2632929

[B18] Meilenger D Fellinger K Bultmann S Rothbauer U Bonapace IM Klinkert WEF Spada F Leonhardt H Np95 interacts with de novo DNA methyltransferases, Dnmt3a and Dnmt3b, and mediates epigenetic silencing of the viral CMV promoter in embryonic stem cells EMBO reports 2009 10 1259 1264 10.1038/embor.2009.201 19798101 PMC2756565

[B19] Jin W Liu Y Xu Sg Win Wj Li Jj Yang Jm Shao ZM UHRF1 inhibits MDR1 gene transcription and sensitizes breast cancer cells to anticancer drugs Breast Cancer Res Treat 2009 DOI: 10.1007/s10549-009-0683-8. 10.1007/s10549-009-0683-8 20037778

[B20] Muto M Kanari Y Kubo E Takabe T Kurihara T Fujimori A Tatsumi K Targeted disruption of Np95 gene renders murine embryonic stem cells hypersensitive to DNA damaging agents and DNA replication blocks J Biol Chem 2002 277 34549 34555 10.1074/jbc.M205189200 12084726

[B21] Muto M Fujimori A Nenoi M Daino K Matsuda Y Kuroiwa A Kubo E Kanari Y Utsuno M Tsuji H Ukai H Mita K Takahagi M Tatsumi K Isolation and characterization of a novel human radiosusceptibility gene, NP95 Radiation Research 2006 723 733 10.1667/RR0459.1 17067204

[B22] Tamblyn L Li E Sarras H Srikanth P Hande MP McPherson JP A role for Mus81 in the repair of chromium-induced DNA damage Mutation Research 2009 660 57 65 19026666 10.1016/j.mrfmmm.2008.10.013

[B23] Hande MP Samper E Landsdorp P Blasco MA Telomere length dynamics and chromosomal instability in cells derived from telomerase-null mice J Cell Biol 1999 144 589 601 10.1083/jcb.144.4.589 10037783 PMC2132934

[B24] Papait R Pistore C Grazini U Babbio F Cogliati S Pecoraro D Brino L Morand AL Dechampesme AM Spada F Leonhardt H McBlane F Oudet P Bonapace IM The PHD domain of Np95 (mUHRF1) is involved in large-scale reorganization of pericentromeric heterochromatin Molecular Biology of the Cell 2008 19 3554 3563 10.1091/mbc.E07-10-1059 18508923 PMC2488286

[B25] Jenkins Y Markovtsov V Lang W Sharma P Pearsall D Warner J Franci C Huang B Huang J Yam GC Vistan JP Pali E Vialard J Janicot M Lorens JB Payan DG Hitoshi Y Critical role of the ubiquitin ligase activity of UHFR1, a nuclear RING finger protein, in tumour cell growth Mol Biol Cell 2005 16 5621 5629 10.1091/mbc.E05-03-0194 16195352 PMC1289407

[B26] Paull TT Rogakou EP Yamazaki V Kirchgessner CU Gellert M Bonner WM A critical role for histone H2AX in recruitment of repair factors to nuclear foci after DNA damage Current Biology 2000 10 886 895 10.1016/S0960-9822(00)00610-2 10959836

[B27] Therman E Susman M Human Chromosomes, Structure, Behaviour and Effects 1993 New York, NY: Springer-Verlag

[B28] Hoffelder DR Luo L Burke NA Watkins SC Gollin SM Saunders WS Resolution of anaphase bridges in cancer cells Chromosoma 2004 112 389 397 10.1007/s00412-004-0284-6 15156327

[B29] Taddei A Maison C Roche D Almouzni G Reversible disruption of pericentric heterochromatin and centromere function by inhibiting deacetylases Nature Cell Biol 2001 3 114 120 10.1038/35055010 11175742

[B30] Peters AHFM O'Carroll D Scherthan H Mechtler K Sauer S Schofer C Weipoltshammer K Pagani M Lachner M Kohlmaier A Opravil S Doyle M Sibilia M Jenuwein T Loss of the Suv39 h histone methyltransferases impairs mammalian heterochromatin and genome stability Cell 2001 107 323 337 10.1016/S0092-8674(01)00542-6 11701123

[B31] Harper JW Elledge SJ The DNA damage response: ten years after Mol Cell 2007 28 739 745 10.1016/j.molcel.2007.11.015 18082599

[B32] Dery U Masson JY Twists and turns in the function of DNA damage signaling and repair proteins by post-translational modifications DNA Repair 2007 6 561 577 10.1016/j.dnarep.2006.12.009 17258515

[B33] Murga M Jaco I Fan Y Soria R Martinez-Pastor B Cuadrado M Yang SM Blasco MA Skoultchi AI Fernandez-Capetillo O Global chromatin compaction limits the strength of the DNA damage response J Cell Biol 2007 178 1101 1108 10.1083/jcb.200704140 17893239 PMC2064646

[B34] Karagiannis TC KN H El-Osta A Disparity of histone deacetylase inhibition on repair of radiation-induced DNA damage on euchromatin and constitutive heterochromatin compartments Oncogene 2007 26 3963 3971 10.1038/sj.onc.1210174 17213813

[B35] Cowell IG Sunter NJ Singh PB Austin CA Durkacz BW Tilby MJ Gamma H2AX foci form preferentially in euchromatin after ionizing radiation PLoS ONE 2007 2 e1057 10.1371/journal.pone.0001057 17957241 PMC2020439

[B36] Ayoub N Jeyasekharan AD Bernal JA Venkitaraman AR HP1-beta mobilization promotes chromatin changes that initiate the DNA damage response Nature 2008 453 682 686 10.1038/nature06875 18438399

[B37] Goodarzi AA Noon AT Deckbar D Ziv Y Shiloh Y Lobrich M Jeggo PA ATM signaling facilitates repair of DNA double-strand breaks associated with heterochromatin Mol Cell 2008 31 167 177 10.1016/j.molcel.2008.05.017 18657500

[B38] Luijsterburg MS Dinant C Lans H Stap J Wiernasz E Lagerwerf S Warmerdam DO Lindh M Brink MC Dobrucki JW Aten JA Fousteri MI Jansen G Dantuma NP Vermeulen W Mullenders LHF Houtsmuller AB Verschure PJ van Driel R Heterochromatin protein 1 is recruited to various types of DNA damage J Cell Biol 2009 185 577 586 10.1083/jcb.200810035 19451271 PMC2711568

[B39] Aucott R Bullwinkel J Yu Y Shi W Billur M Brown JP Menzel U Kioussis D Wang G Reisert I Weimer J Pandita RK Sharma GG Pandita TK Fundele R Singh PB HP1-beta is required for development of the cerebral neocortex and neuromuscular junctions J Cell Biol 2008 183 597 606 10.1083/jcb.200804041 19015315 PMC2582898

[B40] Ziv Y Bielopolski D Galanty Y Lukas C Taya Y Schultz DC Lukas J Bekker-Jensen S Bartek J Shiloh Y Chromatin relaxation in response to DNA double-strand breaks is modulated by a novel ATM- and KAP-1 dependent pathway Nature Cell Biol 2006 8 870 876 10.1038/ncb1446 16862143

[B41] Sun Y Jiang X Xu Y Ayrapetov MK Moreau LA Whetstine JR Price BD Histone H3 methylation links DNA damage detection to activation of the tumour suppressor Tip60 Nature Cell Biol 2009 11 1376 1382 10.1038/ncb1982 19783983 PMC2783526

[B42] Bernstein BE Meissner A Lander ES The mammalian epigenome Cell 2007 128 669 681 10.1016/j.cell.2007.01.033 17320505

[B43] Seligson DB Horvath S McBrian MA Mah V Yu H Tze S Wang Q Chia D Goodglick L Kurdistani SK Global levels of histone modifications predict prognosis in different cancers American Journal of Pathology 2009 174 1619 1628 10.2353/ajpath.2009.080874 19349354 PMC2671251

